# Integration of single-nuclei RNA-sequencing, spatial transcriptomics and histochemistry defines the complex microenvironment of NF1-associated plexiform neurofibromas

**DOI:** 10.1186/s40478-023-01639-1

**Published:** 2023-09-28

**Authors:** Vladimir Amani, Kent A. Riemondy, Rui Fu, Andrea M. Griesinger, Enrique Grimaldo, Graziella Ribeiro De Sousa, Ahmed Gilani, Molly Hemenway, Nicholas K. Foreman, Andrew M. Donson, Nicholas Willard

**Affiliations:** 1 Department of Pediatrics, University of Colorado Anschutz Medical Campus, Aurora, CO USA; 2 Morgan Adams Foundation Pediatric Brain Tumor Research Program, Children’s Hospital Colorado, Aurora, CO USA; 3 RNA Biosciences Initiative, University of Colorado Anschutz Medical Campus, Aurora, CO USA; 4 Computational Biology, New York Genome Center, New York, NY USA; 5 Department of Pathology, University of Colorado Denver, Aurora, CO USA

## Abstract

Plexiform neurofibroma (PN) is a leading cause of morbidity in children with the genetic condition Neurofibromatosis Type 1 (NF1), often disfiguring or threatening vital structures. During formation of PN, a complex tumor microenvironment (TME) develops, with recruitment of neoplastic and non-neoplastic cell types being critical for growth and progression. Due to the cohesive cellularity of PN, single-cell RNA-sequencing is difficult and may result in a loss of detection of critical cellular subpopulations. To bypass this barrier, we performed single-nuclei RNA-sequencing (snRNA-seq) on 8 frozen PN samples, and integrated this with spatial transcriptomics (ST) in 4 PN samples and immunohistochemistry to provide morphological context to transcriptomic data. SnRNA-seq analysis definitively charted the heterogeneous cellular subpopulations in the PN TME, with the predominant fraction being fibroblast subtypes. PN showed a remarkable amount of inter-sample homogeneity regarding cellular subpopulation proportions despite being resected from a variety of anatomical locations. ST analysis identified distinct cellular subpopulations which were annotated using snRNA-seq data and correlated with histological features. Schwann cell/fibroblast interactions were identified by receptor/ligand interaction analysis demonstrating a high probability of Neurexin 1/Neuroligin 1 (NRXN1/NLGN1) receptor-ligand cross-talk predicted between fibroblasts and non-myelinated Schwann cells (NM-SC) and subtypes, respectively. We observed aberrant expression of *NRXN1* and *NLGN1* in our PN snRNA-seq data compared to a normal mouse sciatic nerve single-cell RNA-seq dataset. This pathway has never been described in PN and may indicate a clear and direct communication pathway between putative NM-SC cells of origin and surrounding fibroblasts, potentially driving disease progression. SnRNA-seq integrated with spatial transcriptomics advances our understanding of the complex cellular heterogeneity of PN TME and identify potential novel communication pathways that may drive disease progression, a finding that could provide translational therapy options for patients with these devastating tumors of childhood and early adulthood.

**Supplementary Information:**

The online version contains supplementary material available at 10.1186/s40478-023-01639-1.

## Introduction

Neurofibromatosis type 1 (NF1) is an inherited genetic disorder predisposing patients to tumors in the peripheral nervous system (PNS). This autosomal dominant disorder affects 1 in 3000 at birth [13, 22]. NF1 is caused by a mutation in the *NF1* gene on chromosome 17 coding for the RAS inactivating protein neurofibromin [5]. This increases classic RAS-MAPK pathway signaling, thereby promoting cell proliferation and survival leading to tumor formation. Patients with NF1 suffer from a number of symptoms and signs commonly used for diagnosis that include: hamartomas of the iris, café-au-lait macules, axillary freckling, optic pathway gliomas, osseous lesions and neurofibromas [15]. The focus of this study is on plexiform neurofibroma (PN), tumors causing significant morbidity, and which have the ability to transform into malignant peripheral nerve sheath tumors.

Previously, treatment options were limited for PN and confined to surgery, however, surgical options can have a higher rate of risk compared to patient benefit due to the highly infiltrative nature of the tumors. Additionally, many PN cannot be completely resected because of their locations and many need to have multiple debulking procedures on the same tumors as partially removed PN often regrow [2]. Patients suffer from many forms of morbidities ranging from disfigurations, pain, impaired motor functions, urinary incontinence, obstruction of bowels and life-threatening morbidities such as airway obstruction. These morbidities are often dependent on location of the PN and also rates of changes to tumor volume during progression [10]. Recent advances have focused heavily on the use of RAS/MAPK inhibition, namely the use of mitogen-activated protein kinase kinase (MEK) inhibitors such as selumetinib. Selumetinib was FDA approved following groundbreaking studies showing its effectiveness in PN by the Intramural NIH group [11, 17]. There are, however, certain caveats to MEK inhibition. Most importantly the response to selumetinib is variable in PN. Resistance to the drug has been seen and there is often rebound in the PN after cessation of therapy with substantial short-term toxicities, especially in skin and gut. Although the Phase II data suggested selumetinib can be used for extended periods, there is limited long-term toxicity data. The need for additional biologically driven therapeutic targets is apparent and may involve targeting of different cell fractions. Single-cell RNA-sequencing has been used to identify high risk populations for therapeutic targeting in pre-clinical models. For example, recent studies have used single-cell technologies to drive the design of CAR-T by identifying de novo targets, finding novel target antigens in acute lymphoblastic leukemia with minimum expression in health tissue thereby improving both efficacy and safety [9]. Other studies have used single-cell technologies to outline changes in the immune microenvironment in relapsed B-cell acute lymphoblastic leukemia that influence resistance to therapy, informing new immunotherapy design to combat newly acquired extrinsic regulators [30]. This breakthrough technology is also being used in clinical studies for example a recent trial that used single-cell technologies to identify how leukemia stem cells in acute myeloid leukemia can confer resistance to venetoclax-based therapy, informing the use of alternative therapeutic strategies using azacytidine to combat resistance and benefit patient survival [25, 26]. Thus, strong rationale exists for the use of single cell technology to expand our understanding of PN cellular heterogeneity.

PN development is a complex process starting in non-myelinating Schwann cells (NM-SC) having a homozygous loss of the active neurofibromin protein. The GTPase-activating region is the functional domain of the neurofibromin protein, and without its activity, GTP-bound RAS is not converted to its inactive GDP-bound form. This activates RAS-mediated cell growth through a series of signaling cascades leading to a pro-survival, pro-tumor environment [5]. NM-SC, rather than myelinating Schwann cells, are the key neoplastic element in neurofibroma despite both cell types harboring loss of *NF1*. Homozygosis loss of *NF1* is the key factor leading to rapid cell growth and hyperplasia, but this alone is insufficient for formation of PN which may require cross-talk with other infiltrating cell types [28]. PN have a complex tumor microenvironment (TME), due to cellular sequestering mechanisms involved in tumor formation. An array of cell types are recruited to the area of hyperplasia during tumor formation. Collagen secreting fibroblasts, perineural cells, blood vessels and a high density of degranulated mast cells are all present in these tumors and may play critical roles in maintaining TME and promoting growth. The fast growing NM-SC are surrounded by an inflammatory microenvironment, a scenario shown in other cancers to promote tumor growth and progression [3]. Recent studies have examined the role of immune and stromal cells in the progression of PN and identified NF-κB related predicted cross-talk mechanisms between cell types, predominately in mouse models [16]. Here, we advanced the current understanding of PN using two complementary transcriptomic analyses to chart cellular heterogeneity in human PN. PN are known by histology and earlier single-cell RNA sequencing studies to contain multiple different cell types [1, 16]. These tumors are very fibrous and attempts to disaggregate into single-cell preparations result in the loss of important cell populations in the TME. Therefore, single-nuclei RNA (snRNA-seq), which only requires snap frozen tissue, was used as a viable alternative. snRNA-seq techniques were used to fully categorize and molecularly define the various cellular subpopulations harbored by PN. Secondly, we utilized spatial transcriptomics (Visium) to provide morphological context to molecularly defined cellular heterogeneity based on snRNA-seq which was validated using immunohistochemistry. This combined approach has succeeded in defining heterogeneity [6] and offers a novel and comprehensive view into the in situ environment of this poorly understood tumor. Additionally, aberrant spatial features and gene expression levels were used to identify potential targetable ligand-receptor cellular cross-talk pathways.

## Materials and methods

### Sample details and preparation

Human NF1-associated plexiform neurofibroma samples (Table 1) were collected from surgeries at Children’s Hospital Colorado in compliance with all ethical standards and informed consent (COMIRB-13-1672). Bulk frozen tissue samples were processed using CHAPS-based buffer as previously described that provided nuclei that were not only high in membrane integrity, but also nuclear yield [27]. An additional wash step was utilized to prevent the presence of cell-free RNA that has been shown to confound downstream snRNA-sequencing. Bright field microscopy was employed to determine yield, quality and membrane integrity of single nuclei. Archival formalin-fixed paraffin-embedded (FFPE) material corresponding to 14 PN patient samples were obtained for IHC analyses.

**Table 1:** Description of sample cohort

Sample	Age	Location	snRNA-seq	Spatial transcriptomics
NF02	6	Scalp	Yes	No
NF09	4	Neck	Yes	Yes
NF12	5	Eyebrow	Yes	Yes
NF13	8	Face	Yes	No
NF14	6	Face	Yes	No
NF15	7	Neck	Yes	Yes
NF16	10	Abdomen	Yes	Yes
NF32	17	Face	Yes	No

### Single-nuclei capture, RNA library preparation, and sequencing

Single-nuclei RNA-sequencing (snRNA-seq) was conducted on 8 frozen PN samples that had been snap frozen at the time of surgery and banked at our institution. All PN samples were taken from patients with confirmed Neurofibromatosis Type 1. snRNA-seq allowed the use of bulk frozen samples and the isolation of single-nuclei with superior quantity and quality with low levels of cell-free RNA as demonstrated by quality control measurements included in the platform. Single nuclei generated from PN were analyzed using 10 × Genomics Chromium 3′ gene expression. The single-nuclei RNA-seq was performed at the UC Cancer Center Microarray and Genomics Core, where 4000 nuclei per sample were captured and barcoded to generate cDNA libraries. Libraries were subsequently sequenced using the Illumina NovaSeq6000 platform at approximately 50,000 reads per nuclei. We utilized Chromium Controller in combination with Chromium Single Cell V3 Chemistry Library Kits, Gel Bead & Multiplex Kit, and Chip Kit (10 × Genomics). Transcripts were converted to cDNA, barcoded, and libraries were sequenced on Illumina NovaSeq6000 to obtain 50,000 reads per cell.

### SnRNA-seq data analysis

Raw sequencing reads were processed using CellRanger (10 × Genomics) and Seurat 3.1.0. C Cells from individual subpopulations were reanalyzed separately using Harmony alignment [18]. Presto was used for determining differential expression and marker gene identification [19]. Cellular subpopulations were fully categorized by using Jaccard analysis of PN cellular subpopulation marker genes with data from a single-cell RNA-sequencing mouse sciatic peripheral nerve atlas [31], as well as comparison with data from the benign cutaneous fibroma study [1], and examination of cluster-specific gene ontologies. CellChatDB [4] (version 1.5.0) was used to infer ligand–receptor interactions between and within individual subpopulations in single cell data. This method measures the average expression of ligand and cognate receptor genes for each subpopulation and then calculates the probability and statistical significance of intercellular communications, and identify dominant senders and receiver cell types.

### Visium spatial transcriptomics

Optimal cutting temperature (OCT) compound embedded frozen primary PN samples were sectioned on a Cryostar NX70 cryostat (ThermoFisher) at 10 μm sections and mounted on spatial transcriptomic capture slides (Visium, 10 × Genomics). The optimal tissue section permeabilization time (18 min) was established using preliminary test sections. 4 PN sections were permeabilized and resulting capture sections underwent methanol fixation and were stained with H&E and imaged on an Evos M7000 (ThermoFisher) with brightfield settings. RNA libraries were generated and subsequently sequenced at 70,000 read pairs per spot (Novaseq6000, Illumina).

### Spatial transcriptomics data analysis

Sequencing data were processed with Space Ranger (10 × genomics, v1.2.1), followed by analysis in R using the Seurat (v4.0.1) tool suite. To ensure the number of genes detected were between 50 and 15,000 and less than 50% of UMIs mapped to mitochondrial genes, spots were filtered. SCTransform normalization on each sample and principal component analysis on merged data was conducted, followed by sample integration with Harmony (v1.0) using 30 principal components and theta = 2. UMAP dimension reduction and shared nearest neighbor clustering were conducted on 30 principal components. Space Ranger (10 × Genomics), Seurat [12], Harmony [18] and Clustree [32] packages were utilized to cluster, process, filter and integrate newly generated ST data. Clustering results at different resolution settings were evaluated through Clustree (v0.4.3) visualizations. The Wilcoxon test was used to determine differential gene expression within ST gene clusters. Cell types were defined by (1) Jaccard index calculation of ST cluster marker gene overlap with snRNA-seq markers as conducted previously [6, 7], (2) manual interpretation of ST cluster markers and (3) ontological analysis using DAVID (Database for Annotation, Visualization, and Integrated Discovery: https://david.ncifcrf.gov; version 6.8) to measure enrichment of GOterm Direct gene-sets in ST cluster signatures. ST cluster overlays on H&E image were produced in the Loupe Browser (10 × Genomics).

### Immunohistochemistry

Immunohistochemistry was performed on 5-µm formalin-fixed, paraffin-embedded (FFPE) tumor tissue sections using a Ventana auto stainer. Antigen retrieval was performed by incubation in citrate solution pH 6.0 for 10 min at 110 °C. Slides were treated with primary antibodies for LAMA2 (Abnova (Catalog #: H00003908-M01), 1:1000), SLC22A3 (Novus Biologicals (Catalog #: NBP1-83976), 1:750), PRRX1 (Thermo Fisher Scientific (Catalog#: MA5-26580), 1:200) and SOX10 (Roche (Catalog#: 760-4968), pre-diluted kit) for 32 min at 37 °C. All immunostained sections were counterstained with hematoxylin. Descriptive histological assessment was provided by board-certified pathologists A.G. and N. W.

### Statistical analyses

Statistical analyses were performed using R bioinformatics, Prism (GraphPad), and Excel (Microsoft) software. Statistical significance was defined as *p* ≤ 0.05 for all tests.

## Results

### Single-nuclei RNA-sequencing analysis of NF1-associated plexiform neurofibroma reveals specific non-neoplastic and neoplastic cellular subpopulations

**Fig. 1:**
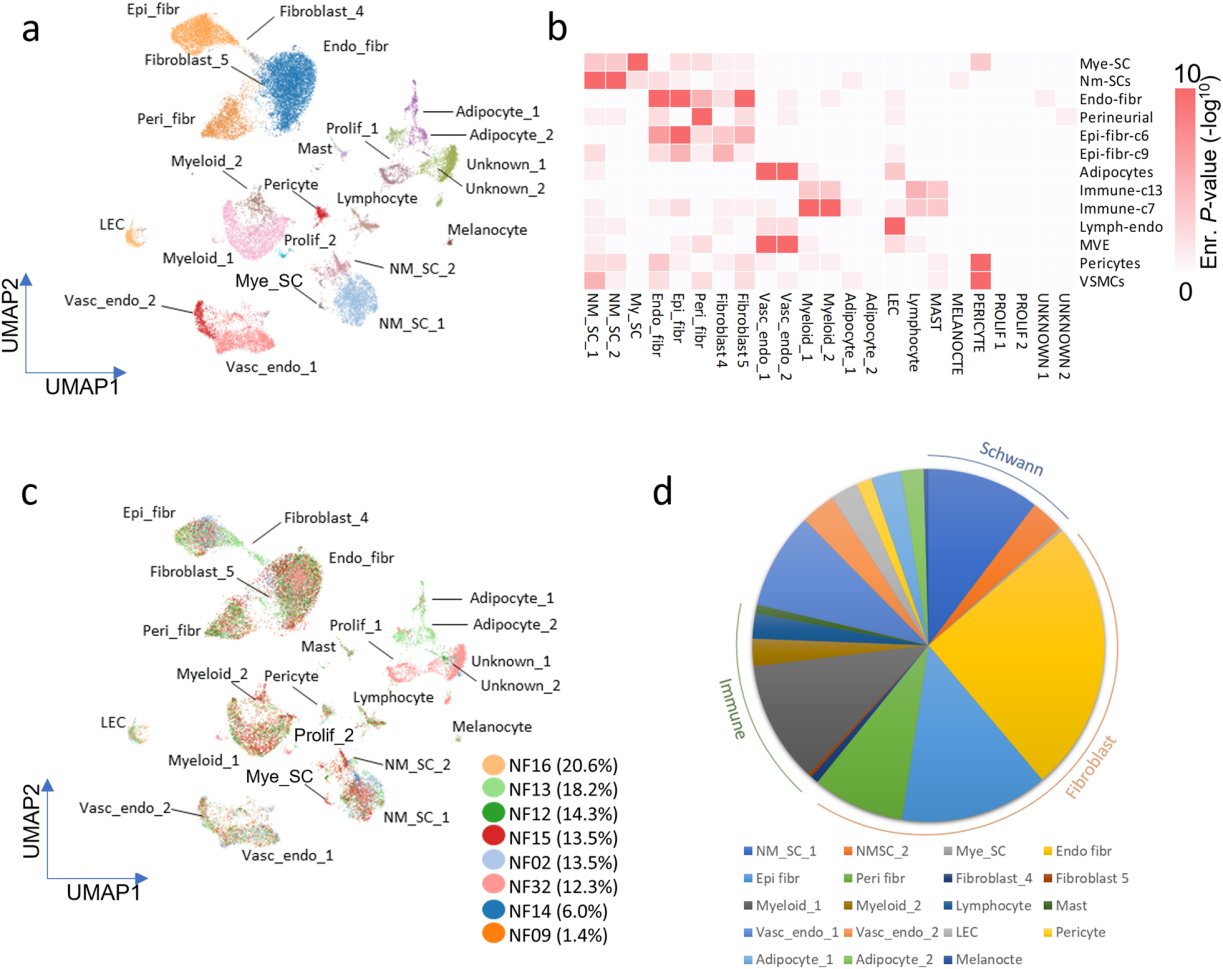
Single-Nuclei RNA-sequencing reveals full characterization of cellular subpopulations harbored by plexiform neurofibromas. **a** Harmony UMAP projection of single-nuclei expression data of 8 PN patient samples on 26,734 nuclei reveals a complex cellular microenvironment with a multitude of infiltrating cell types. **b** snRNA-seq subpopulations were annotated according to cell type deconvolution using Jaccard index analysis which calculates marker gene overlap (displayed as heatmap) with previously published benign cutaneous fibroma scRNA-seq and normal sciatic nerve atlas subpopulation markers. **c** Sample projection overlaid onto harmony UMAP shows most cellular subpopulations shared by all patient samples despite being resected from a variety of anatomical locations. Sample proportions of tumors used to generate UMAP data listed in key. **d** Subgroup proportions suggest a fibroblast rich cellular microenvironment with a strong immune and Schwann cell component. Endo_fibr, Endoneurial fibroblast; Epi_fibr, Epineurial fibroblast; LEC, Lymphatic endothelial cells; Mye_Sc, Myelinated Schwann cell; NM-SC_1 and 2, Non-myelinated Schwann cells 1 and 2; Peri_fibr, Perineurial fibroblast; Prolif_1, Proliferative 1; Vasc_endo_1 and 2; Vascular endothelial 1 and 2

Single-nuclei RNA-sequencing (snRNA-seq) was performed on 8 bulk frozen PN patient samples capturing approximately 4,000 nuclei per sample, a sufficient number of nuclei to provide adequate coverage to report the high levels of cellular heterogeneity found in PN. Cell gene-expression matrices were projected as 2D uniform mani-fold approximation and projection (UMAP) plots (Fig. 1a) which revealed a complex tumor microenvironment. By alignment of PN cellular subpopulation marker genes (Additional file 1) with data from a single-cell RNA-sequencing mouse sciatic peripheral nerve atlas using Jaccard analysis [31], as well as comparison with data from the benign cutaneous fibroma study [1], and examination of cluster-specific gene ontologies (Additional file 2), we were able to fully categorize each cellular subpopulation (Fig. 1b).

We see a distinct separation of non-myelinating Schwann cells (NM-SC) versus myelinating Schwann cells (Mye-SC) (Fig. 1b) based on key genes previously found to distinguish NM-SC (*GFAP, S100*) and Mye-SC (*PRX, MBP, PMP-22*) (Additional file 1) [13]. The tumors harbored a substantial proportion of NM-SC, the putative cell of origin of PN, versus Mye-SC (Fig. 1a). Additionally, we identified discrete endo-, peri- and epineurial fibroblast subpopulations, each featuring distinct gene expression and ontologies (Additional file 1). This fibroblast rich PN tumor microenvironment is consistent with scRNA-seq analysis of benign cutaneous fibromas [1]. The presence of PN endoneurial fibroblasts is consistent with PN being a nerve fascicle-rich tumor, as normal endoneurial fibroblasts are found within the core of the normal nerve sheath where NM-SC reside. The endoneurial fibroblasts expressed genes that distinguish them from other cellular subpopulations: *LAMA2, ABCA10, AL445250, ABCA9* and *EBF2* being the top 5 (Additional file 1). The additional peri- and epineurial fibroblasts are also highly abundant, corresponding to the fibroblasts lining nerve sheaths and exterior of nerve sheaths, respectively. The perineurial cells were defined by *BNC2, SLC22A3, PDZRN4, TENM2* and *SORBS*. Epineurial fibroblasts feature *SLIT2, DCLK1, PRRX1, NAV3* and *RORA* (Additional file 1). The myeloid cellular subpopulations featured key immune signature genes such as *TLR2* and *CD86* and show greatest similarity to the immune-c13 and immune c7 cellular subpopulations in normal sciatic nerve [31] (Fig. 1b) (Additional file 1). Rarer cellular subtypes were captured in the snRNA-seq process such as the mast cell subpopulation which is a diagnostic histological feature of PN.

Interestingly, despite being resected from a variety of anatomical locations (Table 1), PN showed a large amount of inter-sample homogeneity between cellular subpopulations (Fig. 1c). Approximately 50% of the total cells reads were classified as fibroblasts, suggesting that this cell type constitutes a significant fraction of these tumors (Fig. 1d). NM-SC, myeloid and vascular endothelial cells make up the other largest cellular subpopulations (Fig. 1d).

A degree of cellular subpopulation proportion variation was observed within each individual PN sample (Additional file 3). As stated above, the progression of PN relies heavily on different cell types being sequestered to regions of growth, and the variety of cellular subpopulation distribution on a sample-by sample basis reflects this anticipated cellular flux. Some cellular subpopulations such as Adipocyte-1 and -2 and Unknown-1 and -2 (Fig. 1c), were sample-specific, being derived from individual PN (NF13 and NF32 respectively). These were omitted from further study to focus on shared cellular subpopulations rather than sample-specific outliers. Despite variations in cellular distribution, all samples harbored the remaining cell subpopulations identified.

### Spatial transcriptomics reveals morphological heterogeneity across the cellular landscape of PN

**Fig. 2:**
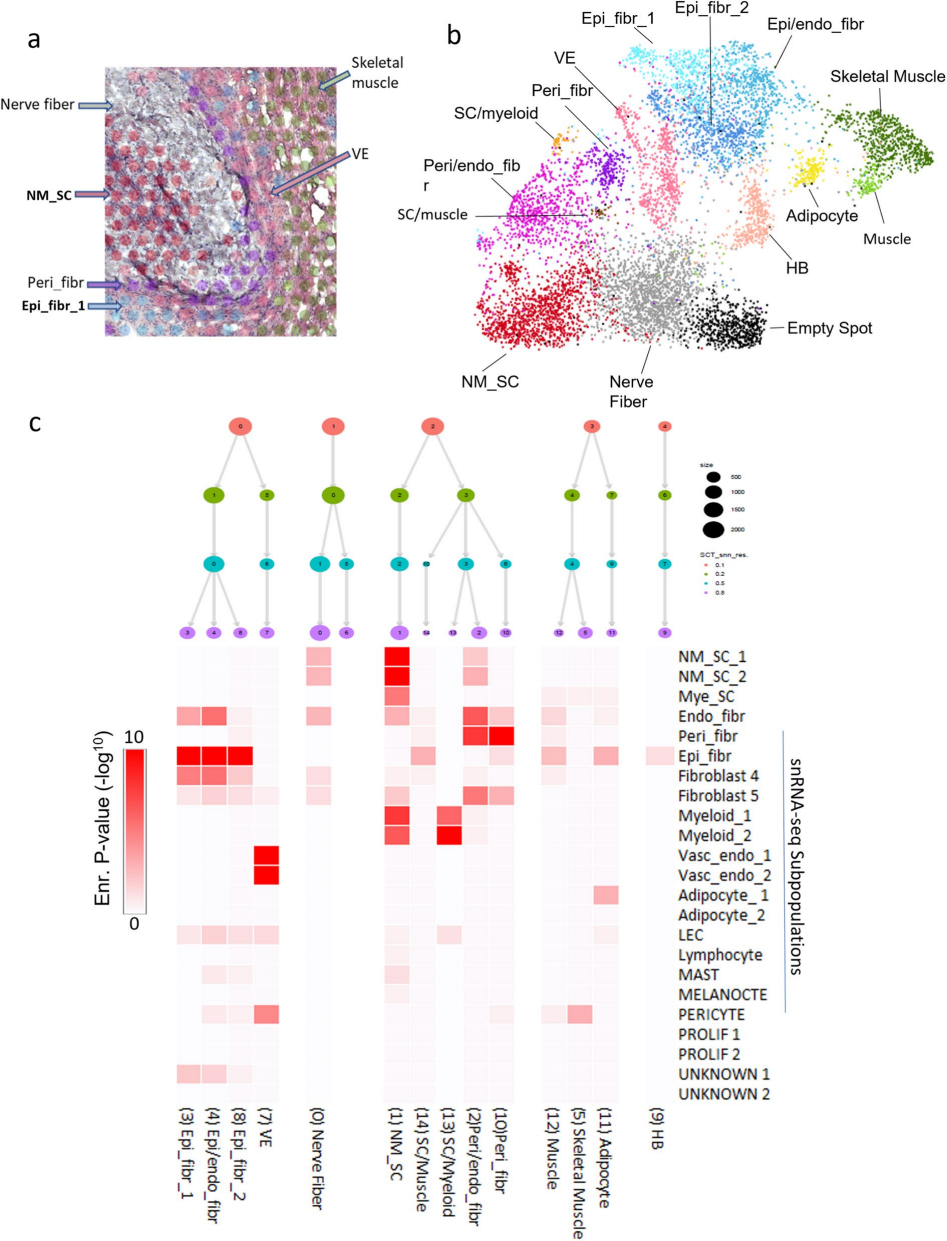
**a** Representative ST sample snapshot highlighting varying numbers of underlying cells per spot range. **b** Sequencing data from 8550 spots in 4 PN samples was aggregated and processed to identify 15 conserved spot clusters using Seurat with Harmony alignment, **c** ST spot clusters were annotated according to cell type deconvolution using Jaccard index analysis, which calculates marker gene overlap (displayed as heatmap) with our single-nuclei RNA-sequencing dataset. Additional annotations were generated from gene ontology searches as well as manual inspection of genelists. ST spot clusters were visualized at different resolutions using Clustree analysis. Abbreviations: Endo_fibr, Endoneurial fibroblasts; Epi_fibr, Epineurial fibroblasts; HB, Hemoglobin; LEC, Lymphatic endothelial cells; Mye_Sc, Myelinated Schwann cells; NM-SC_1 and 2, Non-myelinated Schwann cells 1 and 2; Peri_fibr, Perineurial fibroblast; Prolif_1 and 2, Proliferative 1 and 2; SC, Schwann cell; Vasc_endo_1 and 2; Vascular endothelial 1 and 2

Spatial transcriptomics (ST) (Visium, 10 × Genomics) was applied to 4 frozen primary PN samples adding morphological context to the PN snRNA-seq transcriptomic data. Frozen surgical sections were used and represented a variety of anatomical locations where PN occur (Table 1). ST spots spanned 55 µM which equated to approximately 8 to 12 cells per spot (Fig. 2a). The density of cells beneath each spot was determined by the underlying tissue heterogeneity, cellularity and cell size. Spots are spaced 100 µM apart and therefore cannot replicate or be directly compared to the totality or resolution of snRNA-seq data, but rather represents a transcriptomic sub-sampling of cells. Because of this, exact cell types cannot be determined beneath each spot, but the integration of ST and snRNA-seq allows for spatial context to the snRNA-seq data, complementing it by confirming presence and location of defined cellular subpopulations. Harmony UMAP clustering run on ST showed stable, clearly defined cellular subpopulations, and revealed stable intermixed cellular subpopulations (e.g. Schwann Cell/myeloid) reflecting the known cellular heterogeneity that can occur beneath ST spots (Fig. 2b). Many major cellular subpopulations found in the snRNA-seq characterizations were represented in the ST data, however, detection of rarer subpopulations of PN such as mast cells was not possible (Fig. 2b).

Spot clusters were characterized using two techniques. Jaccard analysis to compare ST cluster marker genelists (Additional file 4) to our previously characterized snRNA-seq cellular subpopulation gene markers. This analysis was paired with Clustree visualization of clustering results from different resolutions [32] (Fig. 2c) that lines up well with the newly defined subpopulations, and separates the designated clusters into clearly defined, stable branches. Many spot clusters were found to contain a spatial intermixing of cellular subpopulations, particularly in the fibroblast subtypes. Endoneurial fibroblasts are seen in spot clusters intermixed with both epi- and perineurial fibroblasts (Fig. 2c, cluster #2, 3,4) unlike epineurial and perineurial fibroblasts that each formed exclusive spot clusters (#5 and #10 respectively). Schwann cell spot clusters were also seen to intermix with both myeloid and muscle cells. Secondly, ontological analysis of ST clusters revealed pathway and cell type profiles enrichment that further clarified cluster designations (Additional file 5). For example, the “Schwann cell/myeloid” spot cluster had the most alignment according to Jaccard analysis with our 2 myeloid snRNA-seq cellular subpopulations (Fig. 2c), but there was also a strong expression of Schwann cell specific marker SOX10 as identified by manual inspection of genelists (Additional file 4). Nerve fibers had light alignment with our NM-SC snRNA-seq cellular subpopulations (Fig. 2c), but the gene marker list was relatively short, and the spot clusters were found within the endoneurium suggesting they represented paucicellular nerve fiber regions.

Of note, the ST spot clusters annotations did not, in all cases, correlate directly to the snRNA-seq data generated. ‘NM-SC’ and ‘Nerve Cell’ designations were assigned to the interior of the nerve fascicle where we would expect to see a majority of NM-SC but also endoneurial fibroblasts (Fig. 2a). The weak endoneurial fibroblast signature present in the NM-SC spot cluster (Fig. 2c) may be due to the dominance of the NM-SC signature. The non-single cell resolutions of the ST spot clusters can also account for this lack of specificity. Other rare cell types such as mast cells that were clearly seen in snRNA-seq data were not present in ST data. The aforementioned “Schwann cell/ myeloid” spot clusters and ‘Endo/epi_fibr and peri_fibr’ annotations also stem from the lack of single-cell resolution inherent in ST technology. The proximity of these varying cell types does however indicate potential direct cellular cross-talk and therefore provide an important biological insight to their roles in disease progression. For example, we see close association and mixing of endoneurial fibroblasts with both peri- and epineurial fibroblasts, suggesting there is a migration of endoneurial fibroblasts into the regions surrounding the nerve fascicles aiding in neurofibromatous proliferation in the epineurial regions. Additionally, we see Schwann cells in proximity to both myeloid cells and muscle cells. This finding suggests that Schwann cells are present outside of the nerve fascicle in the PN disease setting.

### Association of single-nuclei RNA-sequencing and spatial transcriptomic cell types with aberrant nerve fascicle structures characteristic in PN

**Fig. 3:**
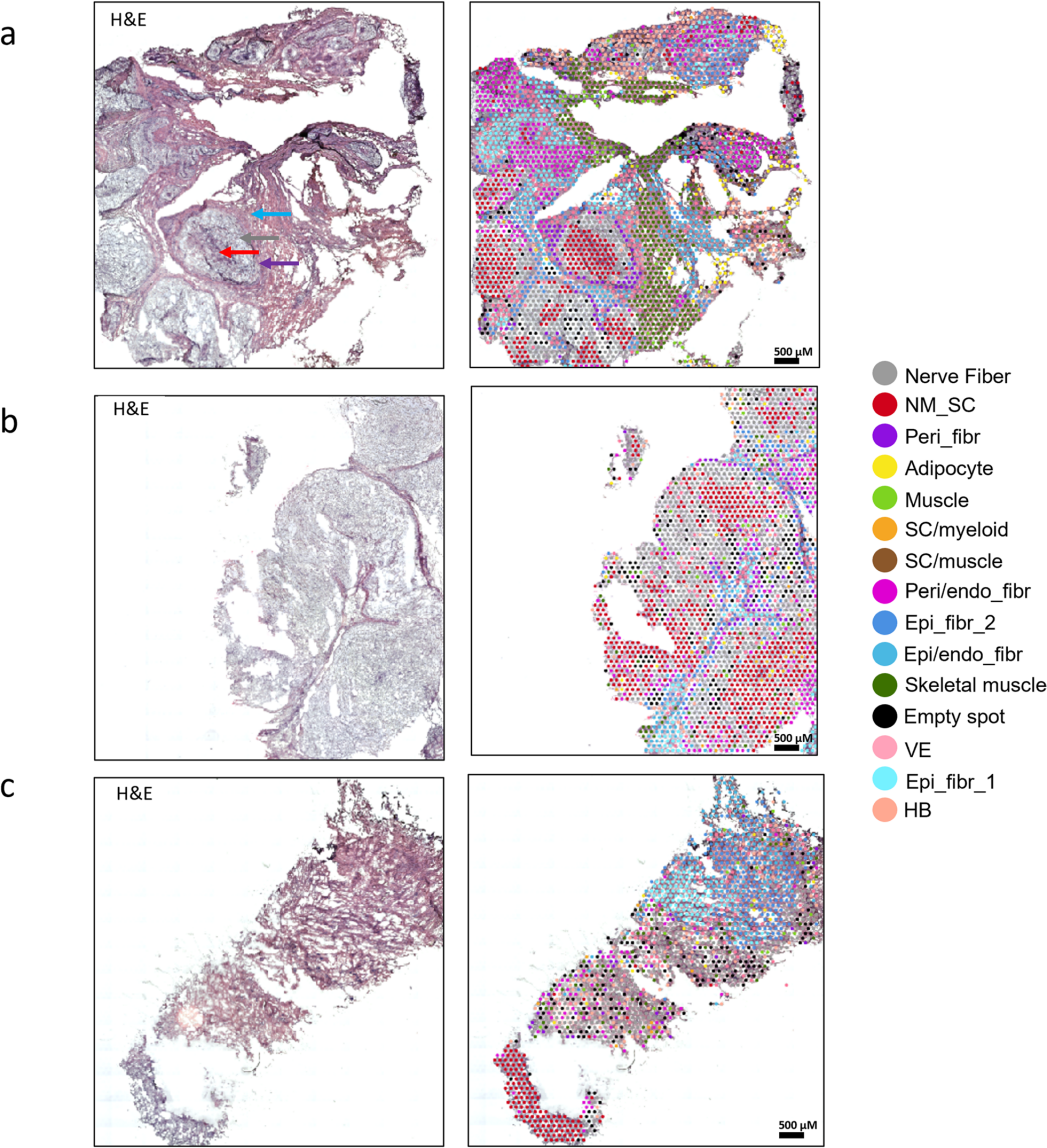
**a**–**c** Representative low magnification images of 3 PN showing classic PN histology by H&E staining (left), including nerve fascicles with surrounding collagen tissue. In left panels (**a**, **b**), the fascicles are surrounded by perineurial fibrous capsule. Between the nerve fascicles is a more prototypical neurofibromatous proliferation. **c** (Panel left) depicts an example of PN architecture that is highly disorganized, with no discernable fascicle features, and highly intermixed cell types. On the right panel, corresponding H&E images with overlayed ST spot clusters revealing extensive heterogeneity of transcriptomic signatures across the TME, with endoneurial regions containing Schwann cells ad nerve cells, flanked by perineurial and epineurial zones containing Schwann cells and CAFs in close proximity surrounded by vasculature and skeletal muscle. Endo_fibr, Endoneurial fibroblasts; Epi_fibr, Epineurial fibroblasts; HB, Hemoglobin; NM-SC, Non-myelinating Schwann cells; SC, Schwann cells; VE, Vascular endothelium

Defined regions of ST spot types align according to the archetypal cellular structures of PN (Fig. 3). The most typical of these features are well-defined, juxtaposed nerve fascicles comprised of myxoid neurofibromatous tissue with scattered ropey collagen. The endoneurial zone is comprised of intermixed Schwann cells and endoneurial fibroblasts flanked (black arrow) by nerve fibers (grey arrow) (Fig. 3a). Each fascicle is surrounded by a well-demarcated perineurial fibrous capsule (purple arrow) (Fig. 3a). The fibrous perineurial capsule is composed of fibroblasts and Schwann cells in close association and surrounded by areas of prototypical neurofibromatous proliferation composed of epineurial fibroblasts (blue arrow), skeletal muscle, adipocytes and areas of vascularization. Strikingly, zones of endoneurial fibroblasts are mixed in with both peri- and epineurial fibroblast, in areas outside of the nerve fascicle (Fig. 3a). Schwann cells are also present in regions outside of the perineurial fibrous capsules, revealing a breakdown of normal nerve architecture in PN (Fig. 3a). Larger nerve fascicles with less epineurial zones were also observed (Fig. 3b), illustrating the diversity of histology typically seen across PN cases. The nerve fibers and Schwann cells are clearly seen within the fascicles. These fascicles are, once again, surrounded by endoneurial fibroblasts mixed with perineurial fibroblasts and regions of epineurial fibroblasts. In some other cases, PN architecture was highly disorganized, with no discernable fascicle features, and highly intermixed cell types (Fig. 3c).

### Orthogonal validation of hallmark gene expression patterns in PN by immunohistochemistry

We identified 3 major fibroblast subpopulations in PN based on snRNA-seq: Endo-, epi- and perineurial fibroblasts. To confirm the presence of these discrete fibroblast subpopulations in PN and their relationship with NM-SC we performed immunohistochemistry (IHC). The fibroblast subpopulations were defined by distinct cluster genelists that distinguish them from one another (Additional file 1). We selected fibroblast subpopulation-specific markers for selection of IHC antibodies from the top the top 5 differentially expressed genes from each—LAMA2 (endoneurial), SLC22A3 (perineurial) and PRRX1 (epineurial). SOX10, an NM-SC specific gene marker was used to identify NM-SCs within the PN samples, and in some cases SOX10 stained NM-SC were found infiltrating all 3 layers of major fibroblast subpopulations.

**Fig. 4:**
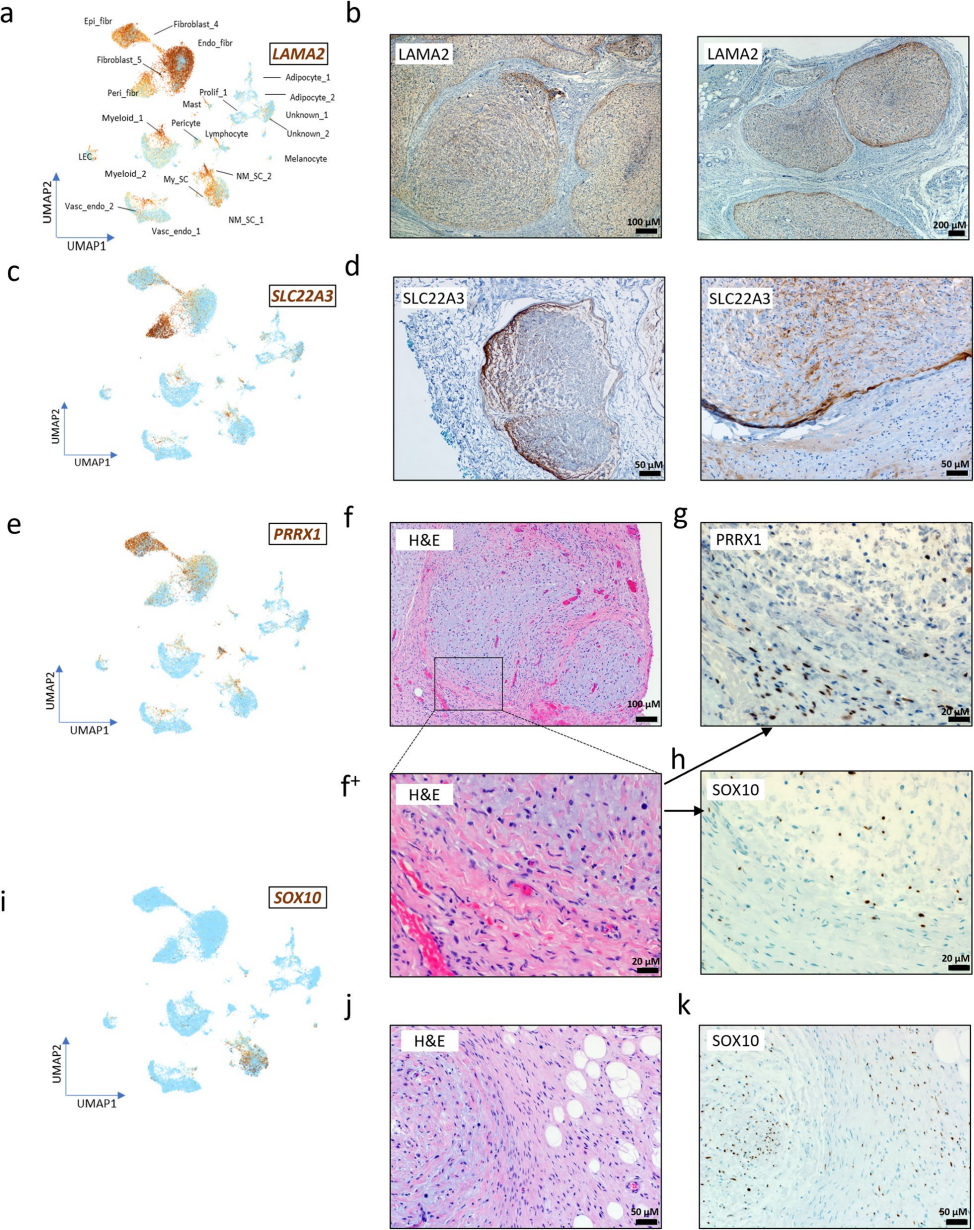
IHC staining was used to validate single-nuclei RNA-seq data. **a** snRNA-seq harmony UMAP expression profile of endoneurial fibroblast marker *LAMA2.* **b** LAMA2 protein expression in 2 PN samples. **c** snRNA-seq harmony UMAP gene expression of perineurial fibroblast marker *SLC22A*. **d** SLC22A3 expression in 2 PN samples. **e** snRNA-seq UMAP expression of epineurial fibroblast marker *PRRX1*. **f** Representative H&E of classic PN morphology. **f**^+^ Zoomed in magnification of region of interest containing endoneurial, perineurial and epineurial zones. **g** PRRX1 protein expression in zoomed in region of interest. **h** SOX10 protein expression in same zoomed in region of interest. **i** snRNA-seq harmony UMAP expression profile of *SOX10*. **j** Representative H&E of second region of classic PN morphology in another patient. **k** SOX10 protein expression in region of interest

*LAMA2* snRNA-seq data showed the highest expression being in the endoneurial fibroblasts with lower expression in the other 4 fibroblast subpopulations, as well as expression in Schwann cells, myeloid, adipocytes and smaller cellular subpopulations (Fig. 4a). By IHC LAMA2 was seen to be expressed primarily in the endoneurium of the oversized nerve fascicles with staining spread into the perineurial zone encapsulating the fascicles. Importantly, there was no LAMA2 staining outside of the oversized nerve fascicles (Fig. 4b). LAMA2 is known to be an extracellular matrix protein that is a major component of the basement membrane.

SLC22A3 IHC was used as a validation stain for perineurial fibroblasts which were found to have a high expression of this gene marker according to snRNA-seq data. Similarly to *LAMA2*, snRNA-seq showed lighter *SLC22A3* expression in the other fibroblast subpopulations, myeloid and Schwann cells (Fig. 4c). The perineurial cells encapsulate the nerve fascicle, and these epithelioid myofibroblasts provide a protective layer around the endoneurium they surround. SLC22A3 acts as a polyspecific organic cation transporter, and by IHC its cytoplasmic staining was predominately seen in the thin perineurial fibroblast layer, with varying amounts of endoneurial staining seen on a sample-by-sample basis (Fig. 4d), once again recapitulating *SLC22A3* expression profiles in our snRNA-seq data (Fig. 4c).

*PRRX1* was found by snRNA-seq to be a strong specific gene marker in the epineurial fibroblast cellular subpopulation with scattered expression in the endoneurial and perineurial fibroblasts as well as the lymphocytes, and to a lesser degree in NM-SC and myeloid cells (Fig. 4e). PRRX1 plays a role in mesenchymal stem cell differentiation, and the collagen-rich epineurium has a structural role in nerve sheath development. To illustrate PRRX1 expression by IHC, we chose a diagnostically relevant zone shown with H&E staining to contain large, misshapen nerve fascicles surrounded by epineurial cells, characteristic of PN (Fig. 4f). Within that region, we zoomed into an area that includes the endoneurium, perineurium and epineurium to show differential expression of PRRX1 between zones (Fig. 4f^+^). Indeed, PRRX1 is expressed predominantly in the epineurial zones surrounding the nerve fascicles (Fig. 4h). This region also contains other cell types such as skeletal muscle, mast cells and vasculature in PN.

To assess the relationship between fibroblast subpopulations and PN NM-SC we used SOX10 IHC. *SOX10* expression was solely seen in the Schwann cell subpopulations in our snRNA-seq data (Fig. 4i). To illustrate differences in SOX 10 expression by IHC, we also used the H&E region of interest used for PRRX1 staining (Fig. 4f^+^). We see clear nuclear SOX10 staining NM-SC contained exclusively inside of the endoneurium (Fig. 4h). Interestingly, we noted SOX10 positive NM-SC existing in epineurial zones in some of our PN samples, as previously observe by ST analysis (Fig. 3a). As an example, another region of a characteristic PN microenvironment containing endoneurial, perineural and epineurial zones as well as adipocytes was chosen where SOX10 NM-SC were not only found in the endoneurium but also scattered throughout the epineurial zones (Fig. 4j, k). This finding suggests that the neoplastic Schwann cells enter regions in between nerve fascicles and infiltrate epineurial zones. Neoplastic Schwann cells are found in close proximity to disease-associated fibroblasts in zones outside of the fascicles, therefore, cellular cross-talk between Schwann cells and fibroblasts could drive progression and histologically-established aberrant nerve fascicle growth that defines PN.

### Ligand-receptor cellular cross-talk prediction identifies potential direct communication pathway between fibroblasts and Schwann cells

**Fig. 5:**
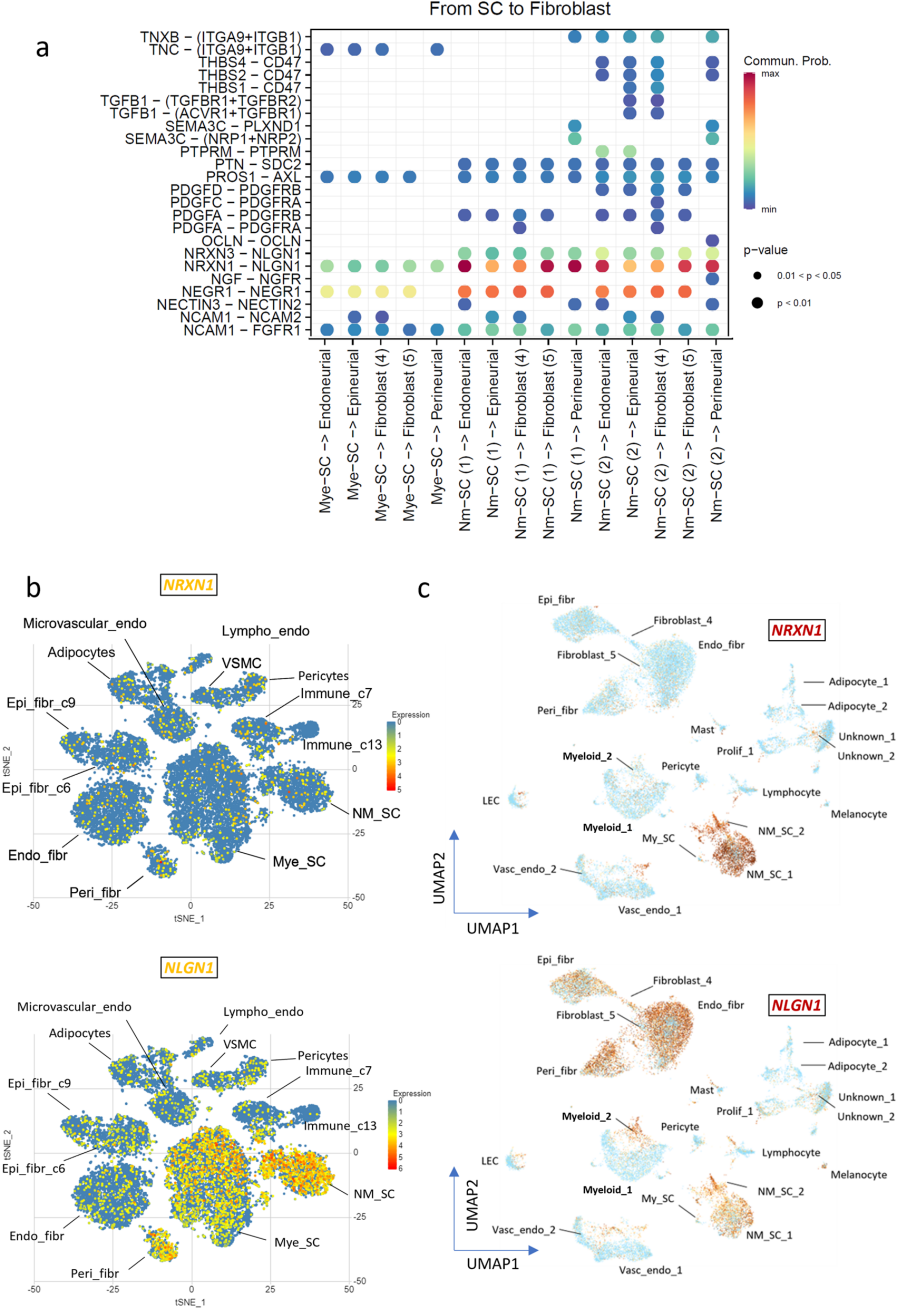
**a** Predicted ligand-receptor cross-talk between Schwann cells and fibroblast subpopulations (directional). **b** *NRXN1* (top panel) and *NLGN1* (bottom panel) gene expression in normal mouse sciatic nerve and **c** corresponding *NRXN1* and *NLGN1* gene expression levels in our PN snRNA-seq data

Analysis of predicted ligand-receptor cell cross-talk pathways between Schwann cells and major fibroblast subpopulations using CellChatDB [4] further strengthened the idea that NM-SC rely on fibroblasts for disease progression. Putative cell-to-cell communication between cellular subpopulations were identified based on overexpression of a ligand by one cell type and overexpression of a cognate receptor by the other cell type. Overexpression of the ligand as an outgoing signal and overexpression of the receptor by a particular cell type is defined as an incoming signal. A strong probability of binding of Neuroligan 1 (NLGN1) to Neurexin 1 (NRXN1) and was predicted, specifically between the three most abundant fibroblast subpopulations and NM-SCs, rather than Mye-SC, respectively (Fig. 5a; Additional file 6), with the highest probability of interaction between endoneurial and perineurial fibroblast-associated NLGN1 and NM-SC-1 associated-NRXN1 (probability of interaction = 0.346 and 0.341 respectively, *p* < 0.0001 for both). NRXN1 and NLGN1 proteins are heavily involved in the formation of glutamatergic synapse formation in synaptogenesis and play a crucial role in neuritogenesis. *NRXN1* expression shows very little expression difference between cell types in the normal Glial Cell Atlas [31], being scattered throughout the various subpopulations, whereas *NLGN1* is highly expressed in NM-SC, Mye-SC and perineurial cells in a normal peripheral nerve environment (Fig. 5b). While *NRXN1* is not normally expressed in normal nerves, it is specifically highly expressed within our NM-SC subpopulations in our snRNA-seq data (Fig. 4c). In fact, *NRXN1* is the top gene that is differentially expressed in our NM-SC cellular subpopulations versus Mye-SC (Additional file 1). Also differing from the normal Glia Cell Atlas *NLGN1* is expressed in predominantly in fibroblast subtypes, particularly endoneurial fibroblasts our PN snRNA-seq data, and to a lesser extent in NM-SC (Fig. 5c). The level of expression of both *NRXN1* and *NLGN1* in NM-SC, as well as the expression of *NLGN1* in endo-, peri- and epi-fibroblasts paired with predicted receptor-ligand interactions, suggests that directional cellular cross-talk between fibroblasts and NM-SC may play a role in the development of PN. This aberrant level of expression of *NRXN1* within NM-SC could indicate a critical pathway for neurite motility and cell-to-cell communication, an observation that is further supported by relatively high expression of fibroblast growth factor receptor-1 (*FGFR1*), that is known to be activated by NRXN1/NLGN1 [8], in the most abundant fibroblast subpopulations previously listed (Additional file 2).

## Discussion

Identification and characterization of cellular subpopulations in plexiform neurofibroma (PN) is essential to reveal underlying biological characteristics of the disease to understand progression and ultimately design novel patient therapy options. Here, we use multiple methods of characterization, single-nuclei RNA-sequencing (snRNA-seq), Spatial Transcriptomics (ST) and immunohistochemistry (IHC), to not only describe the complicated tumor microenvironment (TME) of PN, but also provide morphological context to the transcriptomic data generated. Benign cutaneous neurofibromas had previously been described as fibroblast rich tumors of the peripheral nervous system (PNS) with many forms of infiltrating cell types present according to single-cell RNA-sequencing (scRNA-seq) efforts [1]. Additionally, recent studies have described a variety of different Schwann cell populations in PN via scRNA-seq, and highlighted pathways that may drive stromal cells (fibroblasts) and immune cells to ultimately lead to disease progression [16]. Here we analyzed 8 primary PN samples from a range of anatomical locations using snRNA-seq and spatial transcriptomic technology, which led to a detailed look into the TME of human PN.

We found PN are composed of approximately 50% fibroblasts, with endoneurial, epineurial and perineurial fibroblasts, respectively, being expressed in most abundance within our patient cohort. Schwann cells and especially non-myelinating Schwann cells (NM-SC), the putative cells of origin of PN, make up a substantial percentage of additional cell types which also include myeloid cells, adipocytes and characteristic mast cells known by pathology to infiltrate PN allowing for diagnosis. PN are characterized by regions of over-sized nerve fascicles accumulating in multiple layers of tissue and surrounded by areas of prototypical neurofibromatous proliferation. Indeed, these areas have vascular and skeletal muscle components all represented within our snRNA-seq data. PN samples were shown to have a high level of cellular homogeneity, with only 2 PN found to harbor additional, sample-specific subpopulations. Distribution of cellular subpopulations does vary between samples, but almost all are represented in our 8 PN samples despite being resected from a variety of anatomical locations. Previous studies have described cellular subpopulation flux associated with PN progression [16], and our findings replicate this with additional fully characterized, rare cellular subpopulations.

The integration of ST to our snRNA-seq dataset allowed for full morphological context to our transcriptomic data. ST clusters confirmed the presence of nerve and Schwann cells within the interior of the nerve fascicles, with clear regions of perineurial fibroblasts and surrounding epineurial fibroblasts mixed in with skeletal muscle and vascularization. Interestingly, we see a mix of Schwann cells and both myeloid and muscle cells, indicating that Schwann cells exit the nerve fascicles into fibroblast rich zones in PN. The idea of Schwann cell to fibroblast cell-to-cell signaling could account for PN progression, as the stromal areas surrounding the SC rich nerve bundle interior comprise of zones of tumor growth. Furthermore, during validation of our major fibroblast subpopulations and NM-SC, we note that SOX10 expressing NM-SC are present in epineurial zones between nerve fascicles and in areas of growth.

Our study confirms that a variety of cell types which are normally contained within the endoneurial zones of nerve fascicles, namely NM-SC and endoneurial fibroblasts, are seen to be distributed outside of the nerve fascicles both by spatial transcriptomics and IHC. We show homogeneity in the presence of major fibroblast and Schwann cell subpopulations amongst samples. The use of receptor-ligand prediction algorithms (CellChatDB) allowed us to examine proposed cellular cross-talk mechanisms between major endo-, epi- and perineurial fibroblast cellular subpopulations and NM-SC. CellChatDB provides an unbiased inference of probable cellular cross-talk in single-cell data. It has been used extensively in research to predict likely ligand-receptor interactions [14, 24, 29], some of which have been validated with functional assays [20]. The identification of highly probable and directional Neurexin 1 (NRXN1) and Neuregulin 1 (NLGN1) binding was of particular interest. Our snRNA-seq NM-SC subpopulation is defined most by differential expression of *NRXN1* which is not found to be expressed highly in normal nerve NM-SC cells. Previous studies have confirmed that interaction between NRXN1 and NLGN1 can trigger neurite outgrowth via activation of FGFR1 [8]. NLGN1, specifically, was described as an oncogene shown to be upregulated in NF1-associated breast cancer [21]. Neuronal activity driving solid tumor progression is further supported by neuroligin as a crucial factor for development of NF1-associated optic pathway gliomas, a finding that was confirmed when pharmacological inhibition and genetic loss of NLGN3 resulted in not only blocked progression but also formation of optic gliomas [23]. The aberrant expression of *NRXN1* in PN NM-SC, combined with the presence of its ligand, *NLGN1*, in our all 3 major fibroblast cellular subpopulations is a probable means of communication between putative cells of origin harboring homozygous loss of *NF1* and surrounding fibroblast cells, one based on NRXN1 and NLGN1 binding and proximity to one another. Future studies employing genetic and pharmacologic approaches in mouse models of PN will be an invaluable tool to demonstrate necessity of this NRX1-NLGN1 pathway as a critical determinant of this disease.

Understanding the role of the tumor microenvironment in promoting growth of PN can provide new translational, targetable information to improve therapy for PN and ultimately for prevention of these tumors.

### Supplementary Information

**Additional file 1:** SnRNA-seq cluster marker gene list.

**Additional file 2:** SnRNA-seq cluster ontology list.

**Additional file 3:** SnRNA-seq cellular subpopulation proportions between individual cases.

**Additional file 4:** Spatial transcriptomics cluster marker gene lists.

**Additional file 5:** Spatial transcriptomics cluster ontology list.

**Additional file 6:** CellChat inferred ligand receptor interactions.

## Data Availability

SnRNA-seq and ST data have been deposited in the National Center for Biotechnology Information Gene Expression Omnibus (GEO) database and are publicly accessible through GEO accession number GSE232766 (https://www.ncbi.nlm.nih.gov/geo/query/acc.cgi?acc=GSE232766). A browsable internet resource of the PN snRNA-seq data, including clusters and gene expression for all 8 samples are available at www.pneuroonccellatlas.org.
