# Supplementary material for: Integration of single-nuclei RNA-sequencing, spatial transcriptomics and histochemistry defines the complex microenvironment of NF1-associated plexiform neurofibromas
Source: Acta Neuropathol Commun. 2023 Sep 28;11:158. doi: 10.1186/s40478-023-01639-1 (PMC10537467; doi:10.1186/s40478-023-01639-1)
Supplement: Supplementary file 3 — Additional file 3: SnRNA-seq cellular subpopulation proportions between individual cases. [file 40478_2023_1639_MOESM3_ESM.pdf]

Additional file 3: snRNA-seq subpopulation proportions between individual cases.

category	cell type	subpopulation	NF02	NF09	NF12	NF13	NF14	NF15	NF16	NF32
neoplastic	schwann	NM_SC_1	16.5	16.1	5.5	7.3	22.7	11.7	6.8	4.1
		NM_SC_2	2.6	6.6	1.5	2.1	2.7	4.8	4.3	0.3
non-neoplastic		Mye_SC	0.1	0.5	0.1	0.2	0.3	0.9	0.3	0.3
	fibroblast	Endo_fibr	16.3	29.3	40.3	8.2	25.0	30.4	26.0	16.4
		Epi_fibr	20.7	0.3	9.0	16.7	28.2	6.3	9.8	5.5
		Peri_fibr	2.8	3.2	15.6	3.2	4.1	10.1	12.2	2.2
		Fibroblast_4	0.2	0.0	0.0	3.4	0.4	0.0	0.0	0.0
		Fibroblast_5	0.0	0.0	0.0	1.5	0.0	0.0	0.0	0.0
	immune	Myeloid_1	3.0	19.8	14.9	6.5	0.8	19.7	15.2	2.9
		Myeloid_2	0.5	5.5	1.8	0.9	0.5	4.5	5.1	0.0
		Lymphocyte	0.6	1.3	1.8	6.2	0.4	1.6	1.1	1.0
		Mast	0.4	0.0	0.7	2.3	0.8	0.4	0.1	0.4
	vasc.	Vasc_endo_1	21.7	7.7	2.9	9.4	2.1	5.5	9.4	0.9
		Vasc_endo_2	6.3	4.2	1.0	4.7	0.9	2.4	2.6	0.4
		LEC	5.9	0.0	1.0	2.5	1.1	0.0	3.6	0.7
		Pericyte	2.1	2.6	0.6	2.6	1.4	0.7	0.8	0.8
	fat cells	Adipocyte_1	0.0	0.3	0.3	12.5	0.9	0.1	0.1	0.7
		Adipocyte_2	0.2	0.8	0.4	5.8	0.4	0.2	0.3	5.4
	other	Prolif_1	0.1	0.3	0.1	1.3	0.9	0.2	0.4	16.2
		Prolif_2	0.0	0.0	0.0	0.3	0.0	0.0	0.0	2.2
		Unknown_1	0.1	0.8	0.9	0.8	6.1	0.5	1.7	39.3
		Unknown_2	0.0	0.8	1.5	0.1	0.0	0.1	0.0	0.0
		Melanocyte	0.0	0.0	0.1	1.6	0.3	0.1	0.1	0.4
